# Supplementary material for: Transformative learning of medical trainees during the COVID-19 pandemic: A mixed methods study
Source: PLoS One. 2022 Sep 16;17(9):e0274683. doi: 10.1371/journal.pone.0274683 (PMC9481004; doi:10.1371/journal.pone.0274683)
Supplement: S1 Table — This table provides definitions for the various outcomes and processes of Transformative Learning Theory. (DOCX) [file pone.0274683.s001.docx]

**S1 Table: Definition of Transformative Learning Concepts (10)**

| **Concepts** | **Definitions** |
| --- | --- |
| **Outcomes** |  |
| *Acting differently* | Not just thinking about making changes, but actually doing them as a result of a transformation (e.g., losing weight). |
| *Having a deeper self-awareness* | Becoming aware of your own biases, thoughts, perspectives and how they influence your thoughts and behaviors. |
| *Having more open perspectives* | More open to people or groups of people who are different from you; wanting to understand those differences and asking questions. |
| *Experiencing a deep shift in worldview* | Reassessing early life learning and being able to shift those traditions of learning; shifting from only your own roots (i.e., there is only a Western way of thinking; religious teachings). |
| **Processes** |  |
| **Cognitive Rational Domain** | Personal reflection on one’s own life through concrete, practical, or rational means. |
| *Critical Reflection* | Underlying assumptions of the problem are questioned. It means asking “Why is this important to me? Why do I care about this in the first place?” |
| *Action* | A person has taken action. |
| *Experience* | A person has an experience. |
| *Disorienting dilemma* | The event that happened to a person was disorienting, confusing. |
| *Discourse* | The words that a person used changed the way that person examined a situation either in written or spoken form (e.g., Gettysburg address, Obama offering words to become more involved in schools). |
| **Extrarational Domain** | Personal reflection on one’s own life through abstract, arts-based ways, discussions, or emotions. |
| *Arts based* | Movie, sculpture, photo, piece of art (either doing or seeing creativity). |
| *Dialogue* | The interaction between two people in a conversation (different from discourse because discourse is one-way). |
| *Emotional* | A person experiences emotion, feelings. |
| *Imaginal* | Imagination, putting oneself in an imagined world or fantasy. |
| *Spiritual* | A person has a religious or “higher nature” experience. |
| *Soul work* | Driven by intuition, energy, instincts (e.g., the work of Carl Jung who believed in the archetype of wholeness in all and a transpersonal power that transcends the ego). |
| **Social Critique Domain** | Reflection on how one fits into society and has responsibility for a whole that is larger than him/herself. |
| *Ideology critique* | Learning to recognize how uncritically accepted and unjust dominant ideologies are embedded in everyday situations and practices (i.e., capitalism justifies a system that maintains economic and political inequity). |
| *Unveiling oppression* | Recognizing privilege, oppression in groups of people. |
| *Empowerment* | Feeling self-motivated, becoming stronger and more confident, especially in controlling one’s life and claiming one’s rights (e.g., going to graduate school when it seemed unlikely). |
| *Social action* | Not only recognizing oppression and injustice, but taking action against it. |
