# Supplementary material for: Transformative learning of medical trainees during the COVID-19 pandemic: A mixed methods study
Source: PLoS One. 2022 Sep 16;17(9):e0274683. doi: 10.1371/journal.pone.0274683 (PMC9481004; doi:10.1371/journal.pone.0274683)
Supplement: S2 Table — NOTE: All measures with the same grouping letter are not significantly different from each other (p > 0.05). Measures not sharing the same grouping letter are significantly different from each other (p ≤ 0.05, two-tailed). C, Cognitive Rational process domain; S, Social Critique process domain; E, Extrarational process domain. (DOCX) [file pone.0274683.s002.docx]

**S2 Table: Ranked Means for TLS Processes**

|  | **Mean** | **SD** | **groupings** |
| --- | --- | --- | --- |
| Discourse (C) | 79.60 | 12.4 | a |
| Experience (C) | 74.14 | 15.3 | b |
| Social Action (S) | 72.12 | 17.6 | b |
| Ideology Critique (S) | 69.70 | 19.7 | b |
| Action (C) | 69.09 | 20.0 | b |
| Unveiling Oppression (S) | 68.28 | 22.7 | b,c |
| Dialogue Support (E) | 67.07 | 25.3 | b,c |
| Emotions (E) | 65.45 | 23.9 | c |
| Critical Reflection (C) | 62.02 | 16.2 | c,d |
| Disorienting Dilemma (C) | 55.76 | 25.9 | d |
| Imaginal Soul Work (E) | 53.13 | 20.9 | e |
| Spiritual (E) | 52.53 | 37.8 | e |
| Empowerment (S) | 49.49 | 17.9 | e |
| Arts Based (E) | 29.09 | 25.9 | f |

NOTE: All measures with the same grouping letter are not significantly different from each other (p > 0.05). Measures not sharing the same grouping letter are significantly different from each other (p < 0.05, two-tailed).

C, Cognitive Rational process domain; S, Social Critique process domain; E, Extrarational process domain.
