# Supplementary material for: Transformative learning of medical trainees during the COVID-19 pandemic: A mixed methods study
Source: PLoS One. 2022 Sep 16;17(9):e0274683. doi: 10.1371/journal.pone.0274683 (PMC9481004; doi:10.1371/journal.pone.0274683)
Supplement: Attachment — Submitted filename: Response to Editorial Office PONE-D-21-19528R1.docx [file pone.0274683.s004.docx]

1) Please explain how your submission builds on the published extended abstract from the 2021 Society of General Internal Medicine Annual Meeting and whether any additional information or data are presented in your PLOS ONE submission, that were not presented in the extended abstract.

Authors’ response:

This paper builds on the published abstract presented at the 2021 SGIM annual meeting, reporting several types of additional data as well as additional commentary regarding the findings.

Additional data includes:

1. Interrater reliabilities for the qualitative coding, which helps readers assess the validity of the coding system.

2. Descriptive statistics for all participant characteristics (n, %), all TLS measures (M+SD) and all qualitative codes and subcodes (n, %).

3. Definitions and exemplary quotes for each qualitative code and subcode. None of these (sub)codes were described or reported in the published abstract.

Additional statistical test results include:

4. Tests of ranked means for all TLS outcome and process measures to determine which processes were used more and which outcomes were rated as being higher.

5. Tests of participant group differences for all qualitative codes and subcodes.

Additional interpretation includes:

6. Study strengths and limitations.

7. Implications for practice.

2) Please note that should your paper be accepted, all content including images will be published under the Creative Commons Attribution (CC BY) 4.0 license, which means that they will be freely available online, and any third party is permitted to access, download, copy, distribute, and use these materials in any way, even commercially, with proper attribution. In order to publish any previously copyrighted material, PLOS ONE requires permission from the original copyright holder of the content to publish it under the CC BY 4.0 license.

Please clarify whether the authors have received written permission from 2021 Society of General Internal Medicine Annual Meeting to publish this content specifically under the CC BY 4.0 license and upload the granted permission to the manuscript as a supporting information file.

Authors’ response:

We have attached our reprint order from Springer, the publisher of JGIM.
