# Supplementary material for: Transformative learning of medical trainees during the COVID-19 pandemic: A mixed methods study
Source: PLoS One. 2022 Sep 16;17(9):e0274683. doi: 10.1371/journal.pone.0274683 (PMC9481004; doi:10.1371/journal.pone.0274683)
Supplement: Attachment — Submitted filename: PONE-D-21-19528 Revision 2 Author Response.docx [file pone.0274683.s005.docx]

PONE-D-21-19528R1

Transformative learning of medical trainees during the COVID-19 pandemic: a cross sectional survey

PLOS ONE

Dear Dr. Vipler,

Thank you for submitting your manuscript to PLOS ONE. After careful consideration, we feel that it has merit but does not fully meet PLOS ONE’s publication criteria as it currently stands. Therefore, we invite you to submit a revised version of the manuscript that addresses the points raised during the review process.

1- Please clarify the study design inside the title. I believe its a mix method cross-sectional study, So, please change the title of manuscript.

Transformative learning of medical trainees during the COVID-19 pandemic: A mix method study.

- Authors’ response: We have edited the title as such.

Although you have explain the limitation of study, yet i request to justify your sample size inside the methodology.

- Authors’ response: We have included text in the methods section to address the editor’s concerns. This study was a pilot study (and funded as such) to examine transformative learning during COVID-19 in hopes of informing future work. As such, formal sample size calculations were not completed. Moreover, given recruiting restrictions placed on survey-based COVID-19 research at our institution, we were limited in further recruitment after the present sample size was achieved. Nevertheless, we believe this study holds valuable information that should be disseminated, and as stated, has already informed subsequent work, some of which we have already published.

2-Please prepare the checklist and attached it as supplementary file

<https://journals.sagepub.com/doi/10.1177/1558689819875832>

- Authors’ response: We have included the completed checklist at the request of the editor. However, it appears this checklist was intended for methods papers within the domain of mixed methods (i.e., papers specifically designed with new methodologies). Thus, we do not believe all sections to be relevant for our article (mainly questions 16-20). Nevertheless, we have completed it to the best of our ability.

Please submit your revised manuscript by Oct 06 2022 11:59PM. If you will need more time than this to complete your revisions, please reply to this message or contact the journal office at plosone@plos.org. When you're ready to submit your revision, log on to https://www.editorialmanager.com/pone/ and select the 'Submissions Needing Revision' folder to locate your manuscript file.

Please include the following items when submitting your revised manuscript:

•	A rebuttal letter that responds to each point raised by the academic editor and reviewer(s). You should upload this letter as a separate file labeled 'Response to Reviewers'.

•	A marked-up copy of your manuscript that highlights changes made to the original version. You should upload this as a separate file labeled 'Revised Manuscript with Track Changes'.

•	An unmarked version of your revised paper without tracked changes. You should upload this as a separate file labeled 'Manuscript'.

If you would like to make changes to your financial disclosure, please include your updated statement in your cover letter. Guidelines for resubmitting your figure files are available below the reviewer comments at the end of this letter.

If applicable, we recommend that you deposit your laboratory protocols in protocols.io to enhance the reproducibility of your results. Protocols.io assigns your protocol its own identifier (DOI) so that it can be cited independently in the future. For instructions see: https://journals.plos.org/plosone/s/submission-guidelines#loc-laboratory-protocols. Additionally, PLOS ONE offers an option for publishing peer-reviewed Lab Protocol articles, which describe protocols hosted on protocols.io. Read more information on sharing protocols at https://plos.org/protocols?utm_medium=editorial-email&utm_source=authorletters&utm_campaign=protocols.

We look forward to receiving your revised manuscript.

Kind regards,

Muhammad Shahzad Aslam, Ph.D.,M.Phil., Pharm-D

Academic Editor

PLOS ONE

Journal Requirements:

Please review your reference list to ensure that it is complete and correct. If you have cited papers that have been retracted, please include the rationale for doing so in the manuscript text, or remove these references and replace them with relevant current references. Any changes to the reference list should be mentioned in the rebuttal letter that accompanies your revised manuscript. If you need to cite a retracted article, indicate the article’s retracted status in the References list and also include a citation and full reference for the retraction notice.

[Note: HTML markup is below. Please do not edit.]

Reviewers' comments:

Reviewer's Responses to Questions

Comments to the Author

1. If the authors have adequately addressed your comments raised in a previous round of review and you feel that this manuscript is now acceptable for publication, you may indicate that here to bypass the “Comments to the Author” section, enter your conflict of interest statement in the “Confidential to Editor” section, and submit your "Accept" recommendation.

Reviewer #2: (No Response)

________________________________________

2. Is the manuscript technically sound, and do the data support the conclusions?

The manuscript must describe a technically sound piece of scientific research with data that supports the conclusions. Experiments must have been conducted rigorously, with appropriate controls, replication, and sample sizes. The conclusions must be drawn appropriately based on the data presented.

Reviewer #2: Yes

________________________________________

3. Has the statistical analysis been performed appropriately and rigorously?

Reviewer #2: Yes

________________________________________

4. Have the authors made all data underlying the findings in their manuscript fully available?

The PLOS Data policy requires authors to make all data underlying the findings described in their manuscript fully available without restriction, with rare exception (please refer to the Data Availability Statement in the manuscript PDF file). The data should be provided as part of the manuscript or its supporting information, or deposited to a public repository. For example, in addition to summary statistics, the data points behind means, medians and variance measures should be available. If there are restrictions on publicly sharing data—e.g. participant privacy or use of data from a third party—those must be specified.

Reviewer #2: Yes

________________________________________

5. Is the manuscript presented in an intelligible fashion and written in standard English?

PLOS ONE does not copyedit accepted manuscripts, so the language in submitted articles must be clear, correct, and unambiguous. Any typographical or grammatical errors should be corrected at revision, so please note any specific errors here.

Reviewer #2: Yes

________________________________________

6. Review Comments to the Author

Please use the space provided to explain your answers to the questions above. You may also include additional comments for the author, including concerns about dual publication, research ethics, or publication ethics. (Please upload your review as an attachment if it exceeds 20,000 characters)

Reviewer #2: Thank you for the revisions of this manuscript. This reviewer (#2) also greatly appreciates the analysis done (to satisfy the reviewer’s curiosity) on the group differences for each measure in the other domains to find there was no statistically significant differences between the groups for the scales from the other process domains. The majority of comments were addressed except for the following:

It was suggested to add the months of survey administration and listing some of the qualitative coding within the abstract. The authors may have misunderstood this as their response was pointing out where they are located in the manuscript body. Perhaps there is a word limit to the abstract, but if possible, adding “We used the Transformative Learning Survey in September/October 2020…” would provide context for readers who are skimming the abstract to know that this occurred early in the pandemic.

- Authors’ response: We have added dates in the abstract as requested. We apologize for this oversight. With regard to including codes within the abstract, you are correct that we do not have codes per se. However, we do note our four aggregate codes (responses to the pandemic, dark grey in Table 4). Given that there are upwards of 14 individual codes detailed in table 4, we do not feel that we would be able to accomplish including these in a pithy manner in the abstract while keeping to the 300 word limit, which we have edited in this revision to meet exactly (excluding section headers) after the requested additions by reviewer #2.

________________________________________

7. PLOS authors have the option to publish the peer review history of their article (what does this mean?). If published, this will include your full peer review and any attached files.

If you choose “no”, your identity will remain anonymous but your review may still be made public.

Do you want your identity to be public for this peer review? For information about this choice, including consent withdrawal, please see our Privacy Policy.

Reviewer #2: No

________________________________________
